# Challenges of paediatric palliative care in Romania: a focus groups study

**DOI:** 10.1186/s12904-021-00871-7

**Published:** 2021-11-18

**Authors:** Nadia Pacurari, Eva De Clercq, Monica Dragomir, Anca Colita, Tenzin Wangmo, Bernice S. Elger

**Affiliations:** 1 Institute for Biomedical Ethics, University of Basel, Bernoullistrasse 28, 4056 Basel, CH Switzerland; 2 Department of Pediatric Oncology, The Institute of Oncology, Prof. Dr. Alexandru Trestioreanu, Bucharest, Romania; 3 Department of Pediatric Hemato-Oncology & Bone Marrow Transplantation, Fundeni Clinical Institute, Bucharest, Romania

**Keywords:** Palliative care, paediatric oncology, barriers, Romania, qualitative research

## Abstract

**Background:**

The availability of palliative care facilities for children vary considerably among the European member states. In Romania, a country where health expenditure is among the lowest in Europe, palliative care has been mainly provided by charitable organizations. Despite the high number of children needing palliative care, there is scant literature and research available on paediatric palliative care in Romania. The study explores the viewpoints of various paediatric oncology providers with regard to paediatric palliative care provision in Romania.

**Methods:**

Four mixed focus groups were conducted at four university-affiliated paediatric oncology centres located in three distinct Romanian regions (Bucuresti-llfov, Nord-Est and Nord-Vest). The focus groups were analyzed using thematic coding.

**Results:**

For many healthcare professionals, emotional burden inherent to the profession; unhealthy work-life balance and understaffing were among the biggest barriers to the successful integration of pediatric palliative care. The lack of staff was attributed to a shortage of financial resources, and to the persisting cultural stigma surrounding palliative care and oncology. Also political turmoil was identified as an important obstacle to palliative care implementation.

**Conclusion:**

Significant barriers persist limiting the broader implementation of pediatric palliative care in Romania. In order to render palliative care in pediatric oncology more sustainable, more attention should be paid to the mental health care of healthcare professionals working in this field, to the development of mobile palliative care services and to the emigration of skilled medical staff.

## Background

In Europe, approximately 139,000 children who are in need of palliative care (PC) die each year [1]. PC is a holistic approach addressing physical, psycho-social and spiritual concerns of both patients and their families [2]. According to a recent report of the Council of Europe [3], PC is an essential part of the human right to health and should be integrated in each member state's healthcare system. This means that EU countries should guarantee adequate training on PC for all healthcare professionals (HCPs) and offer respite services for informal caregivers. This is particularly important in the case of paediatric palliative care (PPC) since most children are cared for at home by their parents. According to WHO [4], PC for children with a life-limiting or chronic disease should be integrated at diagnosis alongside curative treatment and continue *irrespective* of the prognosis. However, compared to PC services for adults, facilities for children are not yet widely available and the availability of services, professionals and professional activity vary considerably among the EU member states, with high-income countries being better provided compared to low-to-middle income countries [5].

With the fall of communist governments in the 1990s, there has been an important development of PC services in Central and Eastern Europe and the Commonwealth of Independent States. Still, up until 2009, PC service quality in these countries remained poorly coordinated and unevenly distributed across the territory due to excessive bureaucracy and political instability [6]. Van den Heuvel & Olaroiu [7] report that until the early 2000s expertise on PC, adequate services and information on PC were in shortage in Romania. A more recent study by Enache and colleagues [8] shows that although PC in Romania has developed progressively, the level of services is still below the need and that a national plan for PC was lacking. Other research suggests that in the country PC is provided mostly as inpatient care, with very few funding for home PC [9]. An important exception is the “Casa Sperantei” (House of Hope), a non-profit organization providing free home-based PC for adults and children, with an important educational program in PC for HCPs [10, 11].

In 2014, Romania applied for a loan to the World Bank (2014-2021) to improve access to, quality and efficiency of public health services, including PC care, giving a clear indication of the political intention to advance PC [11]. Furthermore, in 2017 the Romanian Ministry of Health decided to assess the situation of PC in the country [10]. The report shows that PC has been provided mainly by charitable organizations and that more public funds are needed to sustain adequate PC services and educational programs. Another assessment study of the same year [11] reports similar findings, and refers, aside from the financial barriers, also briefly to the misunderstanding surrounding the concept of PC.

Following these assessments, in 2018 the Romanian Ministry of Health approved the Regulation for the organisation and functioning of PC services [12]. The Regulation aims at the progressive development of PC as an integral part of the healthcare system and to develop new PC services, with a specific focus on the development of interdisciplinary PC teams. The Regulation also emphasizes the importance of specialised PC care, including psycho-social, spiritual and bereavement care. It also includes specific references to PC for children: following the WHO indications, it argues that PPC services should be offered 24/7, take place in the child’s home and target the well-being of the whole family.

Even though there have been important improvements in the healthcare for children in Romania, the paediatric healthcare system seems to suffer an enormous deficit of pediatric medical staff due to the emigration of skilled physicians and nurses since 2007 (when Romania joined the EU) [13]. Furthermore, infant mortality in Romania is still the highest in EU and approximately 19,400 children needing PC die each year in the country, representing 14% of the EU pediatric population in need of PC, situating Romania on the second position, after Turkey [1]. Despite these high numbers, there is scant literature and research available on PPC, with some important exceptions [5]. Most studies focus on PC in adults; those that do address PPC are not updated [14] and have taken place mostly in oncology settings, with a specific focus on patient distress [15], pain management [16] and shared-decision-making [17–19]. To our knowledge, no research has been done on the challenges faced by HCPs on how to implement PPC in their practice. Therefore, the present study aims to explore the viewpoints of various paediatric oncology care providers with regard to PPC and to identify perceived barriers to adequate PPC delivery in Romania.

## Methods

The present study is part of a larger project on PPC in Switzerland and Romania, funded by the Swiss National Science Foundation (SNSF) and the Botnar Foundation. The Swiss project, a mixed method study, aimed to understand the end-of-life situation in the Swiss paediatric oncology context and to explore how and by whom decisions regarding the integration of PC were made [20–26]. For Romania, the first part of the project consisted of semi-structured interviews with parents and physicians on shared decision making in paediatric oncology [17–19]. The present study used focus groups (FG) to capture the perspective of different HCPs (physicians, nurses, psycho-oncologists, social workers) from four university-affiliated paediatric oncology centres located in three distinct regions (Bucuresti-llfov, Nord-Est and Nord-Vest) of the country and included also patient representatives. The study received approval from the institutional Ethics Committees of every paediatric centre. For confidentiality reasons, we omitted the names of the four cities in which the university hospitals are located. The first author contacted four oncologists (heads of department of 4 university hospitals) by email and invited them to participate in the study. All of them accepted and they recruited the other participants, following the principle of inter-disciplinarity.

The FGs were carried out between January and June 2017 and took place in three major cities in Romania. They lasted between 70 and 90 minutes. Oral informed consent was sought from all participants. Prior to the start of the FG, the moderator briefly restated the purpose of the overall project and allowed participants to ask questions. The interview guide consisted of questions regarding HCPs personal understanding of PC, existing institutional PPC guidelines, the PPC decision-making process and the family’s role in these decisions, participants’ perceived barriers to the implementation of PPC.

To facilitate thematic analysis [27], FGs were recorded upon consent, then transcribed verbatim in Romanian, and translated into English by the first author. In a next step, the transcripts were transferred into the qualitative analysis software MaxQDA (version 12). In a preparatory phase, two researchers (T.W. and N.P.) used inductive open coding for a sample of one FG. This initial coding was done independently and then the codes were compared. All FGs were later analysed inductively by two researchers (E.D.C. and N.P.). Then, codes identified to form a pattern were grouped under specific themes. Finally, the authors selected specific themes related to barriers and possible strategies for the implementation of PPC, in line with the original aim of the paper.

## Results

A total of 25 professionals participated in the FGs: 13 physicians, 4 psychologists, 4 patient representatives, 2 nurses and 2 social workers. The majority of them were women, and had an average of 14 years of’ experience in pediatric oncology. In three out of four centers, a PC specialist was part of the team. This means that only three professionals (oncologists) out of 25 had specialized PC training. The low number of nurses participating to the FGs was due to dire staff shortages. Analysis with regard to the barriers of PPC integration as perceived by paediatric oncology care providers identified 4 major themes: (1) Healthcare professionals’ concerns about working in a PPC setting; (2) families’ attitudes towards PPC, (3) insufficient availability of and access to PC services (4) unstable political situation. Representative quotes were taken from the various FGs using pseudonyms for the participants as well as the centre to illustrate the results.

### I. Healthcare professionals’ emotional burden of working in a PPC setting

All participants reported that working in the field of paediatric oncology is emotionally challenging and might lead to burnout. Many of them argued that this emotional burden is further exacerbated by the lack of personnel, which results in work overload and an unhealthy work-life balance. As a result, many care providers are reluctant to work in this domain and prefer another specialty, which in turn worsens the understaffing problem.

Nobody wants to work in this field. […] The physicians who could do this specialization *[paediatric oncology],* they don't do it, they don't want it. The reason is the psychological impact, the effort, the kind of medical activity (…) When you need to answer 100 calls, you no longer have time to be with your family (S1, oncologist C1)

I think there are difficult aspects for the staff members because a patient who is in this critical situation (…) occupies time and energy from the staff. The staff is aware that things are not heading in a good direction. Especially in the case of long periods of intensive palliative care, a feeling of tiredness, hopelessness and powerlessness arises (S5, resident physician, C4).

Although the various team members tried to be supportive towards each other, they argued that time was often lacking and they expressed the need for professional psychological support for HCPs who are confronted with dying children. The participants had the impression that in other countries HCPs are better protected and that there is more funding for this type of care. One participant reported doing volunteer work in the PC unit to address the lack of psychological support.

(…) the involvement that we all feel when working with children, there is a kind of interaction and interprofessional support between us, but only when there is time left for that (S4, psychologist, C4).

Psychological assistance is missing (…) It's very tough and I do not think many people want to do this specialization [*psychologist in the paediatric oncology unit*] (S1, oncologist, C2).

This does not happen in other countries. There is a protection system there that I have always admired, but which could only work for us if we were much more numerous (S1, oncologist, C1).

Due to limited financial resources in the hospital, the psycho-emotional support is not considered a necessity. I do volunteering for the palliative care department (S4, psychologist, C3).

### II. Families’ attitudes towards PPC

Many healthcare professionals reported that it is generally more difficult for people to accept the death of a child than that of an elderly person and that parents often blame physicians when things go wrong. Most oncologists argued that parents want to try every type of treatment, also very aggressive ones, to save their children and are unwilling to let their child go. When physicians refuse to continue treatment, they often feel as if parents are accusing them of doing so “on purpose”, as if they have “something against the family” (S4, association representative, C1). Some doctors recognized that their own professional attitude might further nurture parents’ inability to let go. A few participants also claimed that in many cases there was an important stigma against PC.

In our country, it is easier to accept that an elderly patient may have a disease and that maybe nothing can be done. But when we speak about a child, if the evolution is bad, it is generally considered that the physician didn’t want to help (…) most people hang on to anything. I also think that we physicians influence them because of our fighter mentality (S1, oncologist, C2).

There was a family who wanted chemotherapy until the last day... […] it seemed as if they didn’t understand that nothing could be done anymore (S2, oncologist, C3).

In order to improve referral to PC services, some respondents underlined the importance of a referral protocol as they believed that this might be helpful to convince parents. Others highlighted the importance of explaining to families the many benefits of transferring the child to a PC unit (e.g. greater intimacy, specialized care). Still others tried to introduce PC earlier in the course of the child’s illness in order to reduce the stigma surrounding this type of care.

I emphasize that in the palliative care department they will have more intimacy, and specialized care. There are less patients, two nurses, one assistant who can help them and give them more attention compared to the oncology department (…) it takes a lot of work to convince them (…) by informing them already at earlier stages when the child is still in the curative phase, I inform them about the importance and meaning of the palliative care department (S4, psychologist, C3).

Better advertising for PC would be necessary. It also depends on the staff’s capacity to convince. (…) everyone is hoping for a wonder (…) If parents were told from the beginning about the possibility of a different [negative] response… (S6, nurse, C3).

### III. Insufficient availability of and access to PC services

According to most participants, the provision of PC services at home would increase patients’ and families’ quality of life on several levels. However, from the moment patients are discharged from public hospitals, HCPs cannot provide assistance anymore as mobile PC services are mostly lacking in the country. The only institution in Romania offering home care is *The Home of Hope*, which is however present only in a few locations. As a result, most children receive PC in the hospital rather than at home and this constitutes an important burden for the parents. Many families come a long way from home in order to be treated in the hospital. These long distances prevent other family members and friends from visiting and often one parent, usually the mother, needs to abandon her other children and move to the hospital city.

The hospice foundation based in X (name city) provides good quality service (…) But there are other areas where this kind of care does not exist. In our hospital, there are protocols for the inpatient period (pain therapy, etc.), but when patients are discharged, we cannot help them anymore (…) PC should not be provided in the hospital. (…) it would be better to take care of the child in a familiar environment (S1, oncologist, C2).

Mothers always wish to take care of the ill child, but they also want to be at home. (…) mobile PC teams could ensure the social integration of the patients’ families (S5, resident physician, C4).

A few care providers reported that often parents become – out of necessity – PC experts by experience.

Children stay in our hospital for a long time. Thus, the mother gets to learn lots of things about medicine, about the child’s care. […] towards the end, some mothers are really trained in palliative care (S1, oncologist, C4).

Some participants believed that the lack of home care not only negatively affects the life quality of children with PC needs, but also has a negative impact upon families whose child still receives curative treatment. A few care providers regretted the absence of bereavement care for families in most oncology centers.

There are clearly three dimensions that shouldn't be mixed: the patient (and their family) who has just been diagnosed, the patient who is receiving curative treatment and the patient who is having palliative care, because they all bring about different emotions, behaviours and expectations (S4, psychologist, C3).

I believe we should continue to support the family after the child's death, but it is currently not the case (S3, parents’ association representative, C1).

Some participants argued that to overcome the shortage in PC services informal care givers and local communities should be more involved and receive appropriate education.

Local communities should be more active in providing palliative care services to their community. They must make sure that they have specialists, that they have centres. Moreover, it is also a problem of education, I think we should learn already in schools how to take care of a child, of an elderly person, of a person with an advanced stage of disease (S4, parents’ association representative, C1)

Likewise, some respondents believed that priests should be trained to take on more PC care duties, and not just limit their visits to the moment of death because although spiritual support is essential for both the child and their parents, this kind of care is not offered on a continuous basis in most hospitals.

Spiritual counselling is very important. We try to co-opt the hospital’s priest. […] It would be good if a separate duty of PC would be specified in his job description. (S5, psychologist, C1).

They [*the priests*] know how to give the religious services, the mysteries, but they would need some training, just the way the physicians have. (…) people associate his coming only with death, unfortunately.” (S3, parents’ association representative, C1).

### IV Unstable political situation

Various participants argued that due to political changes, in particular frequent succession of different Health Ministers, the staff’s requests and needs in the paediatric oncology departments were not followed up and these requests needed to be constantly renewed. For the same reason, adequate PPC regulation was missing.

We try to discuss something with one minister, who is then replaced, and we need to start all over again (..) The frequent political changes have led to such a frequent turnover of people with decision-making power that every time we asked for something, it was in vain.” (S1, oncologist, C1).

We are still negotiating: “Give me some oxygen” […] Maybe at one moment someone will come and help, maybe new laws will appear, right? […] (S1, oncologist, C3).

Some care providers expressed the urgent need for more financial resources for PC and the willingness on the part of the Romanian government to invest in this type of care.

We have (…) to guarantee the financial resources, to see where and when to distribute them, to equip the department with devices and staff, to sign appropriate contracts with the National House of Health Insurances (S1, oncologist, C3).

## Discussion

In recent years, there has been an increased political interest to integrate PC into the Romanian healthcare system [11]. In order to improve PC provision in the country, a better understanding of the obstacles to its proper implementation is needed. The present study focused in particular on PC in the paediatric oncology setting, a relatively understudied area of research, by exploring the viewpoints of HCPs caring for children with cancer in various centers across Romania.

Results of the study show that for many HCPs the problem of understaffing and the associated issue of unhealthy work-life balance, was one of the biggest barriers to PPC integration. One of the major challenges of the Romanian heathcare system and paediatric healthcare system in particular is the great shortages of staff due to huge outflows of medical personnel to western Europe [13]. It is estimated that yearly about 10% of nurses leave Romania in search for better career opportunities and higher salaries and that currently the public healthcare system has a deficit of almost 40,000 healthcare professionals. Hence it should not come as a surprise that also in the PPC setting, shortage of staff is an important problem. The lack of personnel working in PC was also identified by Mosoiu and colleagues [11] as one of the major weaknesses in their SWOT analysis of PC development in Romania. Interestingly, according to our participants the lack of staff was not only due to a lack of financial resources, but also related to a kind of reluctance to specialize in paediatric oncology due to the emotional burden inherent to the profession. This finding is confirmed by other studies that show that paediatric oncology providers by caring for children with a life-threatening disease are at a heightened risk of burnout and compassion fatigue [28–30]. At the same time, however, we should not ignore the possible persisting cultural stigma around the word “cancer” and “palliative care” in specific. Although cancer, thanks to the many medical improvements, is becoming a chronic disease which can be managed over time, and is thus less stigmatized; studies seem to suggest that in Eastern Europe the word is often still avoided because it is considered as a source of a certain disgrace [19, 31]. Unlike for cancer, the stigma surrounding PC seems to be a more global phenomenon which has been frequently reported in previous studies. In the literature, different solutions have been suggested to deal with this stigma, such as replacing PC with a synonym such as “supportive care”, educating families and HCPs about the benefits of PC, investing in positive word-of mouth by families, making sure that PC is integrated at the beginning of diagnosis and not when curative treatment is no longer in place [22, 32–34].

Our findings thus show that if PC development in Romania aims to be sustainable it should not only focus on more funding but also address healthcare professionals’ aversiveness towards paediatric oncology and PC due to the emotional toll that comes along with it. This means that more attention should be given to psycho-social care, not only for cancer patients and their families, but also for care providers. Our participants reported that this type of care is currently not provided. This should not come as a surprise: previous research shows that psychosocial care interventions are largely missing within the Romanian healthcare system and that training into this type of support (for patients and providers) is underdeveloped in medical, psychology and social work curricula [35, 36]. At the same time, we should not forget that HCPs might be reluctant to seek mental care support out of concern that it might affect their career [37]. Previous research has shown that distress among HCPs who care for dying children and their families is very common and that interdisciplinary interventions such as monthly case-based discussions, interdisciplinary networks and bereavement debriefing sessions might significantly improve HCP’s wellbeing [38]. Unfortunately, in many countries, including high-income ones, this type of support activities is largely lacking due to lack of time and staffing [23].

Professional and intra-team support could help healthcare professionals also to better deal with parents’ often irrational hope for curing the child and the family’s and patient’s mistrust in the medical profession when illness progresses. Research indicates that parent-provider discordance with regard to the prognosis is a common phenomenon in advanced paediatric cancer cases and often the result of HCP’s poor communication skills and their feelings of powerlessness when facing a progressive illness [23, 39].

Although PPC should ideally be offered at home and home care for children is integrated in the Romanian PC regulation of 2018, according to our participants, home care services were largely missing in the country with the result that children were often hospitalized for long time periods and isolated from the rest of the family. Like in other countries [25] the lack of PPC mobile teams is probably a combination of complex and interrelated factors (e.g. the absence of adequate funding, organizational issues and bureaucratic challenges) which in Romania is further exacerbated by the unstable political situation [10] which, according to our respondents, threatens any sustainable PPC development in the country.

### Limitations

Our study has some important limitations. First, the empirical data was collected just before the new PC regulation of March 2018. Hence, the current PPC situation in the country might in the meantime have improved. Still, high-quality and long-term improvements in healthcare need time, even more so in a country as Romania where health expenditure is among the lowest among the EU member states [40] and political instability continues to be a major challenge. Second, the findings are not generalizable to other contexts given the specific paediatric oncology and PC setting in Romania and considering that not all oncology centers in the country were included. Finally, some participants might have been reticent to freely express their opinion due to the fact that the FGs were mixed. Hence, not all barriers might have been identified.

## Conclusion

In recent years, increased efforts have been made to properly integrate palliative care for children in the healthcare system in Romania. Our study shows, however, that significant barriers persist limiting the broader implementation of PPC. In order to render PPC more sustainable, more attention should be paid to the mental health of HCPs working in this field and to the development of mobile PC services. Finally, also the political turmoil in the country and the emigration of skilled medical staff remain an important obstacle.

## Data Availability

The data that support the findings of this study are available from the corresponding author upon reasonable request.
